# Prevalence of Cognitive Decline in Individuals With Diabetes in the United States

**DOI:** 10.7759/cureus.90408

**Published:** 2025-08-18

**Authors:** Siya Patel, Mansi Joshi, Shlok Rawat, Priya Agarwal, Danielle Fastring

**Affiliations:** 1 Medicine, William Carey University College of Osteopathic Medicine, Hattiesburg, USA; 2 Research and Preclinical Sciences, William Carey University College of Osteopathic Medicine, Hattiesburg, USA

**Keywords:** brfss database, cognitive decline, public health education, risk factor management, type 2 diab

## Abstract

Background

Recent research indicates that cognitive decline (CD) may be associated with type 2 diabetes mellitus. Individuals with demographic characteristics significantly associated with CD can be prioritized for earlier screening, which could slow the progression of their CD.

Objective

The objective of this study is to understand the association between diabetes and CD in the US when adjusted for age, race, sex, education, and income.

Methods

Data for this study were derived from the 2021 Behavioral Risk Factor Surveillance System (BRFSS), a compilation of responses from a health-related telephone survey that collects information from participants aged 18 years or older. Participants below the age of 45 years were excluded from this study because they did not participate in CD surveys. The states included were Wisconsin, Ohio, Georgia, the District of Columbia, Tennessee, Texas, Pennsylvania, and Oklahoma. Frequencies and percentages were computed for categorical demographic variables. Bivariate analysis was used to determine whether diabetes and demographic variables were independently associated with CD (p<0.10). Demographic variables that were independently associated (p≤0.10) were included in a multivariable binary logistic regression model, and statistically significant variables (p≤0.05) were retained in the final model.

Results

This study finds that individuals with diabetes are 1.714 (95% CI: 1.709, 1.720) times more likely to report CD when adjusted for race, sex, income, and education.

Conclusion

This study concludes that individuals with diabetes are more susceptible to a decline in cognitive function and therefore CD. A limitation of this study is that the data are self-reported, which may introduce recall bias. Further research is needed to understand the mechanism by which CD occurs in individuals with diabetes.

## Introduction

Cognitive decline (CD) is the progressive deterioration in memory, attention, language, and executive function, and it varies in severity [1,2]. Type 2 diabetes mellitus (T2DM) has emerged as a significant risk factor for CD, contributing to individual morbidity as well as heightened healthcare utilization, including more frequent emergency department visits, home health services, outpatient care, and prescription use [3,4].

Clinical studies reinforce this connection [5,6]. Rhmari Tlemçani et al. found that in a patient population with a median age of 65, 47.5% of patients with diabetes (mean HbA1c of 8%) had a Mini-Mental State Examination (MMSE) score of less than 27/30, indicating cognitive impairment [6]. Antal et al. explored changes in gross brain anatomy over time in patients with T2DM. They found a significant atrophy of gray matter in patients with T2DM [7]. The exact mechanisms bridging DM and CD are not fully understood. Proposed mechanisms are multifactorial and include hypo- or hyperglycemia, insulin resistance (IR), neuroinflammation, glucocorticoid excess, vascular pathology, and neurodegeneration [8]. IR is thought to be associated with increased deposition and decreased clearance of amyloid-beta plaques, resulting in decreased synthesis of the insulin-degrading enzyme system [8]. Chronic peripheral hyperinsulinemia leads to both systemic and cerebral IR, which contributes to brain changes [9]. Cerebral IR alters insulin signaling by modifying pathways such as GLUT4 trafficking, which is essential for neuronal health and memory [9]. Neuroinflammation is proposed to be mediated by microglia and is key to neural regulation [10]. Studies have shown that formyl peptide receptor 1 (FPR1) plays a role in activating microglia. FPR2 has been shown to be increased in microglia in the hippocampus of mice with diabetes [10].

The objective of this study is to evaluate the association between diabetes and CD when adjusted for age, race, sex, education, and income in a US nationally representative sample.

## Materials and methods

The Behavioral Risk Factor Surveillance (BRFSS) is a compilation of health-related questions administered via telephone to collect information from non-institutionalized individuals aged 18 years and older. In 2021, several states participated in the optional modules addressing CD and diabetes. Data were available from participants in Wisconsin, Ohio, Georgia, the District of Columbia, Tennessee, Texas, Pennsylvania, and Oklahoma.

Participants younger than age 45 were excluded from the analysis because the CD module questions were only asked of those 45 and older. The data were weighted according to BRFSS methodological guidelines [11]. The demographic variables analyzed in the study included race, sex, income, age, and educational attainment. CD was determined by a “yes” answer to the question, “Have you experienced confusion or memory loss that is happening more often or is getting worse?” Diabetes diagnosis was determined by a “yes” answer to the question, “(Ever told) (you had) diabetes?” Participants who answered, “Yes, but only during pregnancy,” were excluded from the analysis.

Data were downloaded from the CDC BRFSS portal and imported into IBM Statistical Product and Service Solutions (SPSS, version 29.0.1.0; IBM SPSS Statistics for Windows, Armonk, NY) for cleaning and analysis. Participants with missing values for key variables such as subjective cognitive decline (SCD), diabetes status, and major demographics were excluded. Variables were recoded into appropriate categorical formats for analysis, and all analyses incorporated BRFSS-provided sampling weights to ensure population-level estimates and to account for the survey’s complex design. Frequencies and percentages were computed for all categorical variables, and the sample’s characteristics were summarized via descriptive statistics, with mean and standard deviation or median and range calculated for continuous variables as appropriate. To determine whether diabetes was independently associated with CD, bivariate analyses, including chi-square tests for categorical variables and independent t-tests for continuous variables, were conducted. Variables found to be independently associated at a significance level of p<0.10 were entered into a multivariable logistic regression model and stepped down via backward conditional methods. This provided an adjusted model for the relationship between diabetes and CD while controlling for potentially confounding demographic variables. Adjusted odds ratios (ORs) with 95% CIs were calculated. Variables in the multivariable logistic regression model that remained significant at p<0.05 were considered statistically significant and retained in the best-fitting model.

## Results

Demographic characteristics and self-reported diabetes status by CD status are presented in Table 1. The prevalence of DM was 31.79% in individuals with CD compared to 25.69% of those without DM. Demographic factors such as race, education, income, age, and sex were independently associated with self-reports of CD (p<0.10). The risk of CD increased with age, with a prevalence of 44.70% among individuals aged 65 years or older. Females exhibited a higher prevalence than males. Among racial groups, Caucasians had the highest prevalence of CD at 61.48%. There was no clear trend across income levels; however, individuals earning between $55,000 and $100,000 were at the highest risk of developing CD. Conversely, there was a general trend of decreasing prevalence of CD with increasing levels of education.

**Table 1:** Demographic Characteristics and Self-Report Diabetes Status of Individuals With and Without Cognitive Decline (n = 21,862,752) These are BRFSS 2021 weighted data.

Variables		Cognitive decline	
		Not present, n = 1,017,007 (87.00%)	Present, n = 2,845,746 (13.00%)
Diabetes	Yes	14,985,988 (81.99%)	1,824,736 (68.22%)
	No	3,292,728 (18.01%)	850,255 (31.79%)
Age (years)	45-54	5,493,955 (29.00%)	729,932 (25.69%)
	55-64	5,804,033 (30.63%)	841,338 (29.61%)
	65 or more	7,649,276 (40.37%)	1,269,807 (44.70%)
Sex	Male	8,811,571 (46.34%)	1,289,914 (45.33%)
	Female	10,205,434 (53.67%)	1,555,832 (54.67%)
Race	Caucasian	127,980,260 (67.30%)	17,496,280 (61.48%)
	African American	24,885,270 (13.09%)	4,317,640 (15.17%)
	Other	5,445,410 (2.86%)	1,104,830 (3.88%)
	Multiracial	1,665,740 (0.88%)	299,400 (1.05%)
	Hispanic	25,674,690 (13.50%)	4,341,560 (15.26%)
Income	< $15,000	996,081 (5.24%)	310,548 (10.91%)
	$15,000 to	1,673,506 (8.80%)	420,954 (14.79%)
	$25,000 to	1,967,188 (10.34%)	376,326 (13.22%)
	$35,000 to	1,904,139 (10.01%)	383,791 (13.49%)
	$55,000 to	4,628,489 (24.34%)	544,839 (19.15%)
	$100,000 to	2,969,745 (15.62%)	238,340 (8.38%)
	>$200,000	99,861 (5.24%)	39,183 (1.37%)
Education	Did not graduate from high school	24,880,790 (13.08%)	6,484,020 (22.79%)
	Graduated from high school	52,856,290 (27.79%)	8,805,260 (30.94%)
	Attended college/technical school	54,713,290(28.77%)	8,054,320 (28.30%)
	Graduated from college/technical school or higher	56,575,550 (29.75%)	4,964,360 (17.45%)

Table 2 shows the risk of developing CD in individuals with diabetes when adjusted for demographic factors. This analysis revealed that those with DM were 1.714 times more likely to report CD compared to those without DM, when adjusted for race, income, education, sex, and age (95% CI: 1.709, 1.720). Those who identified as “other races, non-Hispanic” were 1.578 (95% CI: 1.566, 1.590) times more likely to report CD compared to their Caucasian counterparts. Black, non-Hispanic individuals were 1.038 (95% CI: 1.033, 1.042) times more likely to develop CD than Caucasians. Multiracial individuals and Hispanics were found to be at a lower risk of CD compared to Caucasians. With regard to income, as income increased, the risk of developing CD decreased. For example, individuals with a household income of $15,000-$25,000 had 0.951 (95% CI: 0.946, 0.957) times the risk of CD compared to individuals with household incomes less than $15,000. Similarly, those with a household income over $200,000 were 0.171 (95% CI: 0.169, 0.173) times as likely to develop CD compared to those in the lowest income category. With regard to education, compared to individuals who did not graduate from high school, those who graduated from high school, attended college, or graduated from college were at lower risk for CD, with the lowest risk observed among individuals who graduated from college or technical school. Males were 1.020 (95% CI: 1.017, 1.023) times more likely to develop CD than females. Finally, as age increased, the risk of CD also increased. Individuals over sixty-five years of age were 1.041 (95% CI: 1.037, 1.045) times more likely to develop CD than those aged 45-54. Those in the 55-64-year age group were 1.021 (95% CI: 1.016, 1.025) times more likely to have CD compared to individuals 45-54.

**Table 2:** Odds of Self-Report of Cognitive Decline Among Individuals With T2DM When Adjusted for Race, Income, Education, Age, and Sex B: regression coefficient (log odds) for the predictor; df: degrees of freedom; SE: standard error of the coefficient; Exp B: exponentiated B

Predictor	B	SE	Wald χ²	df	P-value	Exp(B)	95% CI for Exp(B) (Lower–Upper Limits)
With diabetes (yes vs. no)	0.539	0.002	100,548.82	1	<0.001	1.714	1.709–1.720
Race (overall)			30,764.72	4	<0.001		
Black, non-Hispanic	0.037	0.002	292.055	1	<0.001	1.038	1.033–1.042
Other race, non-Hispanic	0.456	0.004	13,986.89	1	<0.001	1.578	1.566–1.590
Multiracial, non-Hispanic	–0.311	0.009	1,335.79	1	<0.001	0.733	0.721–0.745
Hispanic	–0.259	0.002	11,531.47	1	<0.001	0.772	0.768–0.776
Income (overall)			177,724.78	6	<0.001		
$15,000–$25,000	–0.050	0.003	293.167	1	<0.001	0.951	0.946–0.957
$25,000–$35,000	–0.307	0.003	10,560.26	1	<0.001	0.736	0.732–0.740
$35,000–$50,000	–0.194	0.003	4,176.27	1	<0.001	0.823	0.819–0.828
$50,000–$100,000	–0.668	0.003	52,642.17	1	<0.001	0.513	0.510–0.516
$100,000–$200,000	–0.980	0.003	79,245.83	1	<0.001	0.375	0.373–0.378
>$200,000	–1.764	0.006	77,697.72	1	<0.001	0.171	0.169–0.173
Education (overall)			34,418.07	3	<0.001		
Graduated high school	–0.390	0.002	27,624.88	1	<0.001	0.677	0.674–0.680
Attended college/technical school	–0.332	0.002	18,770.09	1	<0.001	0.718	0.714–0.721
Graduated college/technical school	–0.465	0.003	27,337.93	1	<0.001	0.628	0.625–0.632
Sex							
Male	0.02	0.002	176.039	1	<0.001	1.02	1.017–1.023
Age (overall)			419.614	2	<0.001		
55–64 years	0.02	0.002	100.179	1	<0.001	1.021	1.016–1.025
≥65 years	0.04	0.002	413.121	1	<0.001	1.041	1.037–1.045
Constant	–1.265	0.003	159,907.55	1	<0.001	0.282	

## Discussion

This study investigated the association between diabetes and CD in individuals 45 and over in the United States of America. The study found that individuals with diabetes were 1.714 (95% CI: 1.709, 1.720; p<0.001) times more likely to report CD than those without diabetes when adjusted for race, sex, income, and education (Table 2). This finding is consistent with Zheng et al. [12], who reported that higher HbA1c levels and diabetes were significantly associated with faster long-term CD in older adults. Over a 10-year period, study participants with diabetes or elevated HbA1c experienced greater declines in global cognition, memory, and executive function compared to those with normal glucose levels [12]. These findings suggest that poor glucose control may contribute to accelerated cognitive aging, highlighting the need for further research on whether optimizing glucose levels can slow CD in individuals with diabetes.

This study also found that individuals with diabetes who identify as other races (non-Hispanics) were 1.578 (95% CI: 1.566, 1.590; p<0.001) times more likely to have CD compared to Caucasians when adjusted for sex, income, and education (Table 2). A study conducted by Hsu et al. [13] found that CD attributable to T2DM was higher in African American participants compared to Caucasian participants. African American study participants with T2DM had higher white matter lesion volumes and performed worse on several cognitive tests than European Americans. However, these racial differences in cognitive performance were significantly reduced after accounting for differences in white matter lesions and were nearly eliminated after adjusting for educational quality, as measured by the Wide Range Achievement Test. These findings suggest that both cerebrovascular burden and disparities in educational quality contribute to observed cognitive differences between racial groups with T2DM. More research is warranted to determine why certain racial groups with diabetes are at a higher risk of developing CD than others. This highlights the need for early preventive measures and interventions before cognitive impairment begins to develop [14].

Our study found that as income increased, the risk of CD decreased when the model was adjusted for age, sex, income, and education. Study participants with diabetes earning an income of $15,000-$25,000 were 0.951 times as likely (95% CI: 0.946-0.957; p<0.001) to develop CD, and those earning greater than $200,000 were 0.171 times as likely (95% CI: 0.169-0.173; p<0.001) to develop CD when compared to those earning less than $15,000 annually (Table 2). This indicates that individuals with diabetes who have lower incomes are at higher risk of experiencing a decline in cognitive function. Guo et al. [15] found that research participants with middle incomes who had diabetes and hypertension had better cognitive status than those with higher incomes. This discrepancy may be due to a lack of access to care for those with lower incomes. Individuals with higher incomes may have greater access to, and quality of, care than individuals with lower incomes [16,17]. These findings emphasize the protective role of financial resources in CD. More research is warranted to better understand the impact of income on the ability to obtain early intervention and screening by healthcare professionals.

Our study found that males were 1.02 times (95% CI: 1.017-1.023; p<0.001) more likely to develop CD compared to females when adjusted for age, race, income, and education (Table 2). Ganguli et al. [18] examined how sex-related factors, including body fat distribution and metabolic markers, contributed to CD in older adults. Although biological sex was not directly identified as a predictor, the researchers used waist-to-hip ratio (WHR), a measure that often differs between men and women, as a proxy for sex-related fat distribution. The findings showed that in individuals with higher WHR, a pattern more commonly seen in men, elevated HbA1c levels were associated with faster CD [18]. In contrast, among those with lower WHR, more commonly seen in women, higher levels of adiponectin were linked to greater CD. These results suggest that sex differences in fat distribution may influence metabolic factors such as hyperglycemia or adiponectin, which are relevant to cognitive aging. Overall, the study indicates that biological sex, through its relationship with body composition, plays an important role in modifying the impact of metabolic health on CD in older adults. The rate at which metabolic and cardiovascular abnormalities develop in diabetics may be different in males and females. This difference may impact the development of CD in patients with diabetes. Cognitive evaluation in both sexes should be conducted on a routine basis to address any cognitive deficits early and slow the decline.

Our study found that as age increases, CD also increases. Individuals older than 65 years were 1.041 times (95% CI: 1.037-1.045; p<0.001) more likely to develop CD when compared to those aged 45-54 years and adjusted for race, income, and education (Table 2). This finding is supported by a study conducted by Rawlings et al. [19], which examined how diabetes and hyperglycemia relate to CD in a population of older adults with a mean baseline age of 76 years. The researchers found that diabetes, especially with poor glycemic control (HbA1c ≥7%) and longer duration (≥5 years), significantly increased the risk of developing mild cognitive impairment and dementia. Importantly, these associations were observed in late life, indicating that older adults with longstanding or poorly controlled diabetes are at higher risk for CD. Implementing early screening protocols and interventions, such as routine cognitive testing by healthcare professionals, may increase awareness in populations most at risk and improve time to intervention [20]. This would potentially decrease the rate of CD in those most at risk.

This study has several notable strengths. It uses data from the BRFSS, a large, nationally representative survey of noninstitutionalized US adults that employs rigorous sampling and weighting procedures to generate population-level estimates. The use of standardized survey questions across participating states enhances comparability, and the inclusion of the CD module enables a population-based examination of an outcome that is often underrepresented in large epidemiologic datasets. The large sample size also provides sufficient statistical power to detect associations and explore subgroup differences.

However, several limitations should be acknowledged. The outcome measure of CD was based on a single self-reported item from the BRFSS CD module. Self-report measures are subject to recall bias, misinterpretation, and social desirability bias, particularly for sensitive or subjective symptoms such as memory problems. A single item may lack the sensitivity and specificity needed to distinguish between transient lapses, normal aging, and clinically significant impairment. Additionally, BRFSS data are cross-sectional, which limits the ability to establish temporal or causal relationships between exposures and outcomes.

Residual confounding is also possible, as certain potential covariates, such as detailed comorbidity profiles (e.g., depression, cardiovascular disease), medication use, cognitive reserve, social support, and access to healthcare, were not available or controlled for in the analysis. The study was further restricted to the eight states that chose to administer the CD module during the study period, which may limit generalizability, particularly if these states differ from non-participating states in demographic composition, healthcare access, or prevalence of cognitive impairment. Finally, BRFSS excludes institutionalized populations, such as nursing home residents, where the prevalence of CD may be higher, potentially leading to underestimation of disease burden.

Future research should employ longitudinal designs with objective cognitive assessments, richer clinical and sociodemographic data, and broader geographic coverage to confirm these findings and clarify the temporal and mechanistic pathways linking diabetes to CD.

## Conclusions

Overall, this study found that individuals with diabetes were more susceptible to a decline in cognitive function and, therefore, CD. These findings underscore the importance of recognizing diabetes not only as a metabolic disorder but also as a potential risk factor for neurocognitive deterioration. Further research is needed to determine the biological and behavioral mechanisms linking diabetes to CD, including the roles of glycemic control, vascular health, and inflammation. In the interim, integrating routine cognitive screening into diabetes management, such as through annual cognitive assessments, may facilitate earlier identification and intervention, potentially mitigating progression and improving long-term outcomes.

## Appendices

**Table 3:** Core Section 7 - Chronic Health Condition

Question text	Variable name	Responses
(Ever told) (you had) diabetes?	DIABETE4	1: Yes
		2: Yes, but if female, told only during pregnancy
		3: No
		4: No, pre-diabetes or borderline diabetes
		7: Don’t know/Not sure
		9: Refused

**Table 4:** Module 18 - Cognitive Decline

Question Text	Variable Name	Responses
The next few questions ask about difficulties in thinking or remembering that can make a big difference in everyday activities. This does not refer to occasionally forgetting your keys or the name of someone you recently met, which is normal. This refers to confusion or memory loss that is happening more often or getting worse, such as forgetting how to do things you have always done or forgetting things that you normally would know. We want to know how these difficulties impact you. During the past 12 months, have you experienced confusion or memory loss that is happening more often or is getting worse?	CIMEMLOS	1: Yes
		2: Yes, but if female, told only during pregnancy
		3: No
		4: No, pre- diabetes or borderline diabetes
		7: Don’t know/Not sure
		9: Refused
